# Immunogenicity of poxvirus-based vaccines against Nipah virus

**DOI:** 10.1038/s41598-023-38010-2

**Published:** 2023-07-14

**Authors:** Emily S. Medina-Magües, Jaime Lopera-Madrid, Michael K. Lo, Christina F. Spiropoulou, Joel M. Montgomery, Lex G. Medina-Magües, Cristhian Salas-Quinchucua, Angela P. Jiménez-Mora, Jorge E. Osorio

**Affiliations:** 1 Department of Pathobiological Sciences, School of Veterinary Medicine, University of Wisconsin, Madison, WI USA; 2 Center for Disease Control and Prevention, Atlanta, GA USA

**Keywords:** Viral infection, Live attenuated vaccines, Adaptive immunity, Mucosal immunology, Vaccines

## Abstract

Nipah virus (NiV), an emerging zoonotic pathogen in Southeast Asia, is transmitted from *Pteropus* species of fruit bats to a wide range of species, including humans, pigs, horses, dogs, and cats. NiV has killed millions of animals and caused highly fatal human outbreaks since no vaccine is commercially available. This study characterized the immunogenicity and safety of poxvirus-based Nipah vaccines that can be used in humans and species responsible for NiV transmission. Mice were vaccinated with modified vaccinia Ankara (MVA) and raccoon pox (RCN) viral vectors expressing the NiV fusion (F) and glycoprotein (G) proteins subcutaneously (SC) and intranasally (IN). Importantly, both vaccines did not induce significant weight loss or clinical signs of disease while generating high circulating neutralizing antibodies and lung-specific IgG and IgA responses. The MVA vaccine saw high phenotypic expression of effector and tissue resident memory CD8ɑ^+^ T cells in lungs and splenocytes along with the expression of central memory CD8ɑ^+^ T cells in lungs. The RCN vaccine generated effector memory (SC) and tissue resident (IN) CD8ɑ^+^ T cells in splenocytes and tissue resident (IN) CD8ɑ^+^ T cells in lung cells. These findings support MVA-FG and RCN-FG viral vectors as promising vaccine candidates to protect humans, domestic animals, and wildlife from fatal disease outcomes and to reduce the global threat of NiV.

## Introduction

Nipah virus (NiV), a *paramyxovirus* from the genus *Henipavirus*, is a zoonotic pathogen that poses a severe global threat to human and animal health^1^. NiV is an enveloped single-stranded RNA virus encoding six structural and three nonstructural proteins, with fusion (F) and glycoprotein (G) membrane-anchored proteins that are the key antigens for NiV vaccine development^2^. Its natural hosts and wildlife reservoirs are *Pteropus* fruit bats, including *P. lylei*, *P. medius*, *P. vampyrus*, and *P. hypomelanus* species^3–6^. The first NiV infection was reported in Malaysia in 1998, followed by outbreaks in multiple countries throughout South and Southeast Asia, including recent cases reported in India, from May to June 2018^7,8^. Infected bats can transmit the virus to various species such as humans, pigs, horses, cats, dogs, and other domestic animals through contact with bat body fluids or the consumption of partially eaten fruits from infected bats^9^. In humans, NiV infection has been associated with the consumption of horse meat, close contact with pigs and horses, proximity to other infected humans, and ingestion of bat-contaminated date palm sap^10–12^.

The NiV bat reservoir host experiences no symptoms. In contrast, human infections range from asymptomatic to fatal encephalitis and severe respiratory disease, with a fatality rate between 40–75% ^9^. In the absence of an available NiV vaccine, supportive care, and the use of the antiviral ribavirin in patients with acute encephalitis have reduced mortality by up to 36%^13^. In swine, NiV infection can cause neurological and respiratory symptoms such as a hacking cough and muscle spasms, reaching a mortality rate as high as 40%^14^.

Because of its broad host range, various modes of transmission, and high fatality rate, NiV has been categorized as a priority pathogen and prompting vaccine development. In this regard, various NiV vaccine strategies have been developed, including subunit vaccines, recombinant virus vectors, virus-like particles (VLP), and mRNA platforms^15^. However, these approaches mainly focus on protecting human health, making them fall short of addressing a One Health-based vaccine approach, a collaborative effort to protect all facets of human, animal, and environmental health. Moreover, few NiV vaccine candidates have tested mucosal delivery methods, significantly reducing their practical application in wild and domestic animal populations, ideal for suppressing the virus before wide-scale outbreaks occur.

Poxviruses are double-stranded DNA viruses that belong to the *Poxviridae* family. Several characteristics make them an excellent antigen delivery platform for recombinant vaccine candidates. For instance, they can accept large foreign DNA fragments, thus allowing them to co-express several foreign antigens^16^. Also, poxviruses replicate in the cytoplasm of host cells, which eliminates the risk of potential integration into the host genome. Additionally, they can infect via mucosal and dermal routes and induce specific cell-mediated and humoral immune responses against transgenes^17^.

Modified vaccinia virus Ankara (MVA) is a highly safe poxvirus used in humans during the smallpox eradication program^18^. It has been evaluated as a delivery platform in vaccine candidates, expressing bacterial, parasites, or viral antigens and inducing protective immune responses^19–21^. Another safe and effective poxvirus vector is raccoon poxvirus (RCN). RCN was first isolated from the upper respiratory tract of apparently healthy raccoons in North America^22^. Recombinant RCN vaccines have been proven safe and effective when administered to various species like bats, domestic cats, piglets, sheep, prairie dogs, chickens, and non-human primates^23–29^.

In this study, we constructed two vaccine candidates, MVA-FG and RCN-FG, that co-express the NiV F and G proteins. We characterized the in vitro expression of these recombinant viruses and then quantified their humoral and cellular immune responses in mice. Furthermore, animals were vaccinated using multiple immunization routes to assess immunogenicity through mucosal delivery, a cornerstone for cost-effective, practical large-scale vaccine campaigns of the NiV reservoir host species.

## Materials and methods

### Cells and viruses

Chicken embryonic fibroblast cells (CEF; Charles River, Cat. No.: 10100807), baby hamster kidney cells (BHK-21; American Type Culture Collections (ATCC; Cat. No.: CCL-10), and African green monkey (*Cercopithecus aethiops*) kidney epithelial cells (Vero; ATCC, Cat. No.: CCL-81) were cultured in Dulbecco's modified Eagle media (DMEM; Corning, VA, Cat. No.: 90-113-PB), 5% fetal bovine serum (FBS; Gemini Bio-Products, Cat. No.: 50-753-2978), and antibiotics (1X Antibiotic-Antimycotic; Gibco, Cat. No.: 15240062) and incubated at 37 °C and 5% CO_2_. Wild-type (WT) MVA was obtained from BEI Resources (Manassas, VA, Cat. No.: NR-727); while the MVA-GFP strain used in this study was previously described by inserting green fluorescent protein (GFP) into the hemagglutinin gene of WT-MVA^30,31^. RCN wild-type (RCN-WT) was obtained from ATCC (Cat. No.: VR-838). Viruses were stored at − 80 °C until use. Viral titers were determined by a standard plaque assay^32^ and expressed as plaque-forming units per milliliter (PFU/mL).

### Expression cassette designs

**Figure 1:**
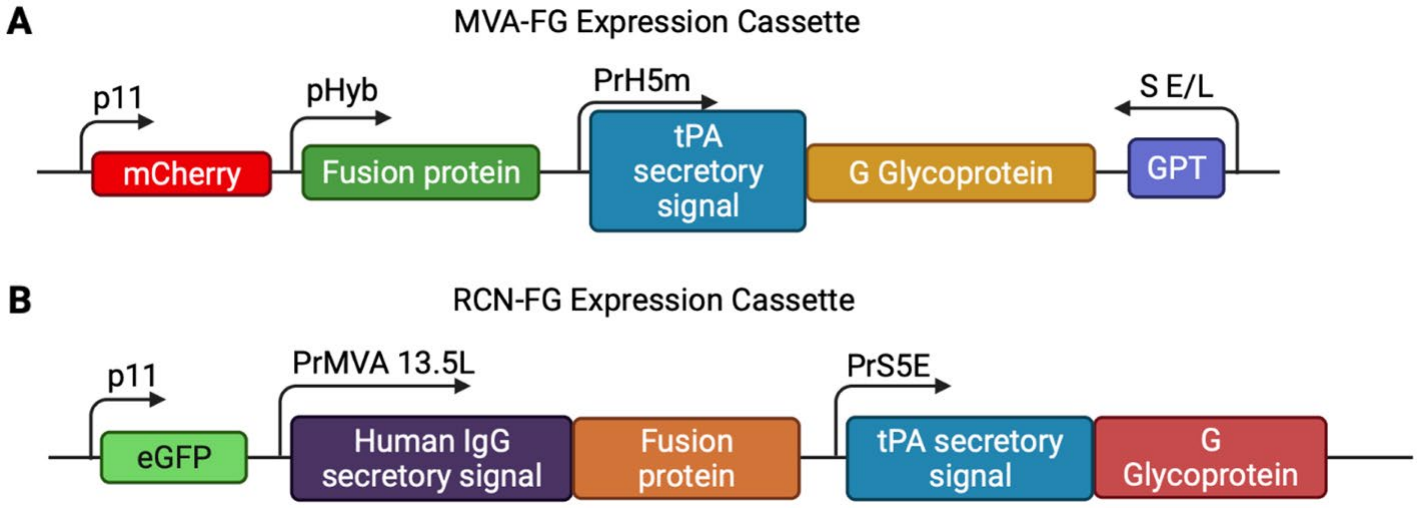
Cassette designs for MVA-FG and RCN-FG vaccine constructs. (**A**) MVA-FG expression cassette consisted of the F gene under the control of the pHyb promoter, followed by the G gene fused with the tPA secretory signal and driven by the PrH5m promoter from vaccinia virus. Genes for fluorescent protein mCherry and GPT were also included for selection of recombinant clones. The F and G sequences were taken from the 2004 NiV Bangladesh outbreak (GenBank: AY988601). This cassette was inserted as a gene knock-out for a parental MVA-GFP virus. (**B**) RCN-FG cassette was designed to contain the F gene fused with the human IgG secretory signal under the control of the PrMVA13.5L promoter. Similarly, the G gene was fused to the tPA secretory signal and expressed using the PrS5E promoter. The eGFP gene was included for the selection of recombinant viruses. The F and G sequences were taken from the 2018 NiV outbreak in Kerala, India (GenBank: MH523642). Figures were created with BioRender.

The MVA gene cassette (Fig. 1A) contained the F and G protein sequences from the 2004 Bangladesh strain of NiV (GenBank: AY988601). The strong pHyb promoter was placed in-frame with the full-length F gene containing a natural secretory signal to drive its expression, and the modified early/late PrH5m promoter (H5 gene from vaccinia virus) was used to control the expression of the G gene in frame with the tissue plasminogen activator (tPA) secretory signal^33^. The mCherry fluorescence marker (GenBank: ANF29837) and the xanthine guanine phosphoribosyl transferase (GPT) selection marker (*Escherichia coli*, GenBank: 75205729) were included to facilitate the selection of recombinant viruses.

For the RCN expression cassette (Fig. 1B), the F and G protein sequences from the 2018 NiV isolated during an outbreak in Kerala, India (GenBank: MH523642) were used. The F protein was fused with the human IgG secretory signal after removing the natural virus-derived secretory signal and expressed under the control of the PrMVA13.5L promoter (naturally occurring promoter in vaccinia virus; GenBank: MT227314). Similarly, the G protein was fused with the tPA secretory signal, and the hybrid PrS5E promoter was used to control its expression. The inclusion of the eGFP protein (GenBank: MN623123) permitted an easy distinction between wild-type (no fluorescence) and recombinant (green) viruses.

Protein sequences were back-translated, codon-optimized for vaccinia virus, and synthesized commercially (GenScript, Piscataway, NJ, USA). The MVA gene cassette was inserted into MVA-GFP as a gene knock-out for GFP as described previously^30,31^, while the RCN gene cassette was inserted upstream to the thymidine kinase (TK) gene of the RCN-WT.

Importantly, gene sequences used in the MVA-FG vaccine were derived from virus isolated from a human infected with NiV whereas gene sequences used in the RCN-FG vaccine were isolated from a bat infected with NiV. While sequences had high homology (99% amino acid homology in the F protein and over 95% amino acid homology in the G protein), difference sequences were used based on the target species for both vaccines and the limited data on how minor amino acid changes can affect antibody binding.

### Production of recombinant viruses

In vitro production and amplification of recombinant viruses were performed in the BSL-2 lab. PCR-amplified DNA for each expression cassette was purified using a Zymoclean Gel DNA recovery kit (ZYMO Research, Irvine, CA, USA, Cat. No.: D4002). PCR amplicons were then encased in a lipid coat using FuGENE^®^ HD reagent (Roche Diagnostics, Indianapolis, IN, USA, Cat. No.: E2311) and used to transfect MVA-GFP infected CEF cells and RCN-WT infected Vero cells as described previously^34,35^. Cells expressing mCherry (MVA) and GFP (RCN) were selected under a fluorescent microscope for five rounds of selection until complete plaque purification was confirmed through PCR^35^. A single plaque isolate was amplified to generate a high titer seed as previously described and underwent Sanger sequencing at the University of Wisconsin-Madison (UW-Madison) Biotechnology Center to ensure genetic stability^35^.

### Immunofluorescence assay

A 12-well plate of either BHK-21 (MVA) or Vero (RCN) cells was infected with one multiplicity of infection (MOI) = 1 PFU/cell with MVA-FG and RCN-FG. The MVA-GFP and RCN-WT viruses were used as their corresponding negative controls. At 24 h (hr) post-infection (p.i.), cells were fixed with cold methanol/acetone (1:1) for 15 min (min) for intracellular protein expression or 4% paraformaldehyde for extracellular protein expression and then washed with 1X phosphate buffer saline (PBS). Cells measuring intracellular protein expression were permeabilized with 0.25% Triton X-100 in 1X PBS for 15 min at room temperature (RT) and included 0.05% Triton X-100 in the following antibody incubation and washing solutions. Cells were then washed with 1X PBS and blocked with 3% bovine serum albumin (BSA) in 1X PBS for 1 h at RT. After blocking, the primary antibody (in-house, 1:1000 dilution) in 3% BSA and 5% FBS was added to cells and incubated at RT for 2 h in the dark. Cells were then washed with 1.5% BSA in 1X PBS three times for 10 min and overlaid with the secondary antibody (goat-anti mouse Alexa Fluor IgG (H + L) highly cross-adsorbed plus 488, 1:3,000 dilution) in 3% BSA and 5% goat serum (Thermo Fisher Scientific, Waltham, MA) at RT for 1 h in the dark. Cells were washed with 1X PBS for 10 min three times and visualized under a fluorescent microscope (AMG EVOSfl, Thermo Fisher Scientific, Waltham, MA, USA).

### Ethics statement

All mouse studies followed the guidelines described in the National Institute of Health's *Guide for the Care and Use of Laboratory Animals*^36^. All animals and their facilities were under the authority of the UW-Madison School of Veterinary Medicine and supervised by the UW-Madison Research Animal Resource Center. Protocols were approved by the UW-Madison Institutional Animal Care and Use Committee (IACUC; approval # V006189) and animal studies were conducted in accordance with the ARRIVE guidelines.

### Mouse studies

**Figure 2:**
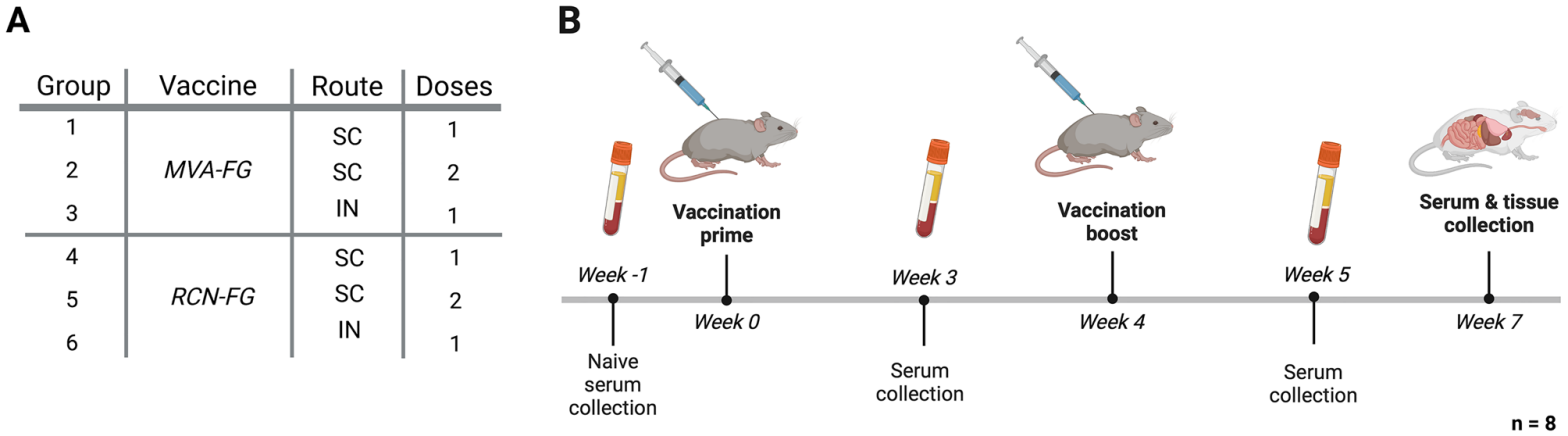
The inoculation, serum, and tissue collection schedule of BALB/c and AJ mice vaccinated with MVA-FG and RCN-FG vaccines. (**A**) Animals were divided into 6 groups, with 3 groups receiving the MVA-FG vaccine (BALB/c) and the remaining 3 groups receiving the RCN-FG vaccine (AJ). (**B**) Vaccines were given SC with a prime, SC with a boost, or IN with a prime. After a naive serum collection (week -1), mice were primed and boosted 4 weeks later with a dose of 10^8^ PFU of MVA-FG or RCN-FG subcutaneously or intranasally. Serum was collected on weeks 3, 5, and 7 to assess humoral immune responses via ELISA and neutralization assays. After humanely euthanizing the animals 7 weeks after prime, spleen and lung cells were collected to measure cytokine-secreting cells via ELISpot. Figures were created with BioRender.

While hamsters are typically used as a model for NiV animal studies, mice were chosen due to ease of handling and as challenge studies were not performed, a species susceptible to Nipah virus was not needed. BALB/c and AJ mice (4-week-old mixed sex) were purchased from Jackson Laboratory (JAX, Sacramento, CA, USA) and housed in a specific-pathogen-free room at the BSL-II Animal Health and Biomedical Sciences building per UW-Madison husbandry protocols. Mice were anesthetized prior to vaccination and blood collection via isoflurane inhalation in a closed-chamber system. Mice were divided into six groups, with eight mice of mixed sex per group (Fig. 2B). After an acclimatization period of 7 days, three groups received the MVA-FG vaccine (BALB/c mice), while the remaining three groups received the RCN-FG vaccine (AJ mice). Groups received one dose via subcutaneous injection (SC) or intranasal (IN) for each vaccine or two doses via SC 28 days apart (Fig. 2A). Viruses were diluted in 1 mM Tris–HCL, and mice were given a dose of 10^8^ PFU for each immunization (50µL per mouse). At weeks 3, 5, and 7 after prime a submandibular blood puncture was performed using a 4 mm animal lancet. Samples were collected in Microvette^Ⓡ^ 500 Z-Gel (Sarstedt, VWR, PA, Cat. No.: 103218-893), kept at RT for 15 min, and centrifuged at 10,000*g* at 20 °C for 5 min. Serum was allocated and stored at − 20 °C for ELISA and neutralization assays. After euthanizing the animals 7 weeks after prime, spleen and lung cells were collected to assess the level of F and G-specific cytokine-secreting cells via ELISpot. Daily observation for any changes in health status by UW-Madison veterinary car staff and weighing three times per week for 2 weeks were carried out after vaccination to monitor changes in the animal’s body condition.

**Figure 3:**
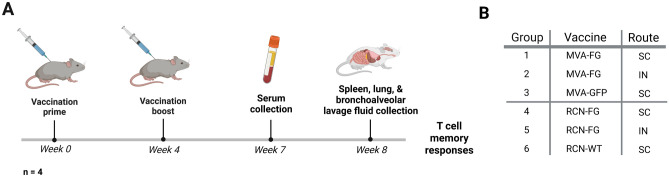
The vaccination, serum, and tissue collection schedule of BALB/c and AJ mice evaluated for T cell memory phenotypic responses, lung-specific antibody generation, and antibody titers in IN boosted mice. (**A**) All mice were primed and boosted 4 weeks later with a dose of 10^8^ PFU of MVA-FG via SC and IN injection, MVA-GFP via SC, RCN-FG via SC and IN injection, and RCN-WT via SC injection. At 7 weeks post-prime, serum was collected to assess anti-F and G antibody titers. For T cell memory evaluation and lung-specific antibody titers, spleen, lung, and bronchoalveolar lavage fluid (BALF) was collected 4 weeks after boost. (**B**) Animals were divided into 6 groups, with 2 groups receiving the MVA-FG vaccine (BALB/c), one group receiving MVA-GFP (BALB/c), 2 groups receiving the RCN-FG vaccine (AJ), and the remaining group receiving RCN-WT (AJ). Figures were created with BioRender.

For flow cytometry studies, vaccines were prepared as described previously, and two doses of 10^8^ PFU for each immunization were given four weeks apart (Fig. 3A). BALB/c mice were divided into three groups, with four mice per group receiving MVA-FG via SC and IN, and MVA-GFP via SC. AJ mice were similarly divided and received RCN-FG via SC and IN, and RCN-WT via SC (Fig. 3B). Serum was collected 7 weeks after prime to assess the humoral immune response of boosted IN groups via ELISA. At 8 weeks post-prime, T cell memory responses were measured in spleen and lung cells and a bronchoalveolar lavage was performed to evaluate the level of F and G specific IgG, IgA, and IgM antibodies in lung tissues.

### Enzyme-linked immunosorbent assay (ELISA)

Immulon 2HB high-binding ELISA plates (Thermo Scientific, Waltham, MA, Cat. No.: 3455) were coated with 100 µg/well of either NiV fusion protein (gF) exodomain (Native Antigen, Kidlington, Oxford, UK, SKU: REC31632) or NiV glycoprotein G (gG) exodomain (Native Antigen, Kidlington, Oxford, UK, SKU: REC31637) diluted in Carbonate-Bicarbonate buffer (Sigma, Saint Louis, MO, Cat. No.: C3041) and incubated overnight at 4 °C. Plates were blocked in either 5% non-fat dry milk (for NiV-G) or 2% BSA (for NiV-F) in PBS-T for 1 h at 37 °C. Naive serum was used as a negative control, and antiserum produced in-house was used as a positive control. Serum samples were diluted in their respective blocking buffers with 5% FBS using three-fold dilutions (1:50 primary dilution) and incubated at 37 °C for 1 h. Serum and BALF samples were incubated with goat anti-mouse IgG (H + L) peroxidase conjugate (Thermo Fisher Scientific, Waltham, MA, USA, Cat. No.: 31430). BALF samples were also incubated with a goat anti-mouse IgA HRP secondary antibody (Thermo Fisher Scientific, Waltham, MA, USA, Cat. No.: 62-6720) and a goat anti-mouse IgM (Heavy chain) HRP secondary antibody (Thermo Fisher Scientific, Waltham, MA, USA, Cat. No.: 62-6820). Secondary antibodies were diluted in their respective blocking buffers with 5% goat serum (1:2,000 dilution) and incubated for 1 h at 37 °C. 1-Step ABTS substrate solution (Thermo Fisher Scientific, Waltham, MA, USA, Cat. No.: 37615) was added, and plates were incubated at RT for 15 min followed by a 1% SDS stop solution (Promega, Madison, WI, USA, Cat. No.: V6551). Plates were read at 405 nm and 650 nm no more than 30 min after visualizing. Reciprocal values of the endpoint dilution were considered positive if they measured greater than the mean value of a naive serum sample plus three times the standard deviation per plate.

### Neutralization assay

For neutralization assays, serum collected during the vaccination study was sent to the US Centers for Disease Control and Prevention (CDC) in Atlanta, Georgia, USA. Serum samples were heat-inactivated at 56 °C for 30 min before being diluted in two-fold dilutions in duplicate (1:40 to 1:5,120). Diluted serum was incubated with 100–150 TCID_50_/well of the Bangladesh NiV strain isolate 810398 (GenBank accession MK673564.1) for 1 h at 37 °C in the BSL-4 and then transferred into confluent Vero cells^37^. Wells were examined for cytopathic effects (CPE) after 6 days p.i. Reciprocal dilution titers were considered neutralizing at the lowest dilution in which both duplicates showed no CPE. Naive sera from BALB/c and AJ mice were tested as negative controls, and rabbit anti-Hendra soluble G serum was used as a positive control (neutralizing up to a reciprocal dilution of 640)^38^. Anti-Hendra G serum was used as a positive control due to its ability to elicit neutralizing antibody responses against both Nipah and Hendra viruses^39^. Viruses were back-tittered in duplicate for virus stocks to confirm intended inoculum amount.

### Spleen, lung, and BALF harvesting

Spleens were removed from freshly euthanized mice, connective tissue was removed, and were rinsed with Hanks balanced salt solution (HBSS, Sigma Aldrich, Darmstadt, Germany, Cat. No.: H9394) in a 35 mm culture dish. Lung tissue was minced in a 35 mm culture dish and digested for 30 min with digestion buffer (Collagenase D (Sigma Aldrich, Darmstadt, Germany, SKU: 11088858001), DNase I (New England Biolabs, Cat. No.: M0303S), FBS, 4 mM glutamine, 25 mM HEPES, and antibiotics in HBSS). Spleens and lungs were crushed using the plunger of a 3 cc syringe and filtered through a 70 µm cell strainer with 1X PBS. Cells were centrifuged at 300*g* for 10 min at 4 °C. The supernatant was removed, and cells were resuspended in a red blood cell lysis buffer (0.85% NH_4_Cl and 0.23% Tris in water) and incubated for 5 min. Cells were centrifuged at 300*g* for 10 min at 4 °C. Cells were resuspended to 3 × 10^6^ cells/mL in CTL-Test™ media (CTL, ImmunoSpot, OH, USA, Cat. No.: CTLT-010) supplied from single-color murine TNF-ɑ and double color murine IFN-γ/IL-2 ImmunoSpot^®^ kits from Cellular Technology Ltd. (CTL, ImmunoSpot, OH, USA).

Following humane euthanasia, mice were pinned, and the neck was disinfected with 70% ethanol. An incision was made at the neck, and skin was pulled back to expose the trachea. A 20 G catheter needle was then inserted above the cartilage rings of the trachea to a depth of 0.5 cm. A 1 mL syringe loaded with 1% FBS in Roswell Park Memorial Institute medium (RPMI) was injected into the lungs, and the aspirated fluid was collected in a 15 mL conical tube. After repeating this process three times, the BALF fluid was centrifuged at 800*g* for 3 min at 4 °C. The supernatant was collected and stored at − 20 °C until analysis via ELISA.

### ELISpot IFN-γ, IL-2, and TNF-α analysis

Spleen and lung cells were added in duplicate with 300,000 cells/well to high-protein-binding PVDF 96-well plates primed with murine IFN-γ/IL-2 capture solution or TNF-ɑ capture solution according to the manufacturer's protocol. Purified NiV-F (Native Antigen) and NiV-G (Native Antigen) antigens were added at 0.2 µg/well in duplicate and incubated at 37 °C for 48 h. Plates were then washed, incubated with corresponding cytokine detection solutions, and developed according to the manufacturer's protocol. Spots were scanned with ImmunoSpot software (Version 7.0.15.2) and counted using the open-source software Viridot^40^.

### T cell memory assessed by flow cytometry

Splenocytes and lung cells were counted and seeded at around 2 × 10^6^ cells/well into 96-well round-bottom plates. Plates were centrifuged at 800*g* for 3 min at RT, and the supernatant was discarded. For each tissue type and animal, the cells were stimulated with 200 µL/well of an F protein-peptide pool (13-mers with 2 amino acid overlap, ThermoFisher), a G protein-peptide pool (13-mers with 2 amino acid overlap, ThermoFisher), a cell stimulation cocktail (TONBO, Cat. No.: TNB-4975) as a positive control, or dimethyl sulfoxide (DMSO)/RPMI-complete medium (Lonza, Cat. No.:BE12-115F) as a negative control. Plates were incubated for 5 h at 37 °C, 5% CO_2_. After incubation, plates were centrifuged at 800*g* for 3 min at 4 °C and washed with 1X PBS where supernatant was discarded. A 1:1000 live/dead cell stain (Aqua-Dye, ThermoFisher) diluted in 1X PBS was mixed with cells and incubated for 30 min at 4 °C in the dark. Cells were then washed with FACS buffer (1X PBS, 0.5% BSA, 0.1% sodium azide) and centrifuged at 800*g* for 3 min at 4 °C, where the supernatant was discarded. Surface staining antibodies (Supplementary Table 1) were added and incubated at 30 min at 4 °C. Cells were then washed with FACS buffer, and cells were fixed and permeabilized (BD Cytofix/Cytoperm, BD Biosciences, Cat. No.: 554722) for 30 min at 4 °C. Cells were then washed with Permwash, and cells were resuspended in 200 µL of FACS buffer, transferred into a FACS tube, and read using a BD LSRFortessa flow cytometer (BD Biosciences, San Jose, CA, USA).

## Flow cytometry gating strategy

Compensation was done using UltraComp eBeads Compensation Beads (ThermoFisher, Cat. No.: A10344) and an ArC Amine Reactive Compensation Bead Kit (Thermo Fisher Scientific, Cat. No.: 01–2222-42). Data were analyzed using FlowJo software (BD Biosciences, TreeStar, Ashland, OR, USA) and an automated flowClean FlowJo plugin^41^. Gating strategies (Supplementary Fig. 1) excluded dead cells using a cell viability stain (Aqua Dye), doublet exclusion via forward scatter height (FCS-H) and forward scatter area (FCS-A) dot plot and selected for proliferating CD4^+^ and CD8α^+^ T cells using a forward (FSC) and side scatter (SSC) dot plot. Live, CD4^+^ and CD8α^+^ lymphocytes were then gated positive at the FITC axis and the BUV395 axis, respectively, in a FITC-BUV395 dot plot. Similar approaches were used to identify populations of CD4^+^ and CD8α^+^ T cells expressing cell surface markers. Samples were excluded from analysis for tissue samples yielding non-viable cell isolates.

## Statistical Analysis

Statistical analysis was performed between vaccine delivery groups for antibody titers using a one-way Kruskal Wallis Analysis of variance (ANOVA) and assessed using a Dunn’s multiple comparison test upon discovery of significant differences. Flow cytometry analysis was conducted using a one-way ANOVA and a Tukey’s multiple comparison test after the discovery of significant differences. All data were analyzed with GraphPad Prism version 9.1.1 software for Mac, GraphPad Software, San Diego, California USA, www.graphpad.com. P-values < 0.05 (alpha) were considered statistically significant.

## Results

### *Production and *in vitro* characterization of viral vector vaccines*

The presence of the F and G genes, including the mCherry and eGFP genes, with their corresponding promoters and regulatory sequences, was confirmed via PCR and cell culture for both MVA-FG and RCN-FG virus constructs. DNA sequencing indicated no genetic alterations in the final virus stocks (data not shown).

**Figure 4:**
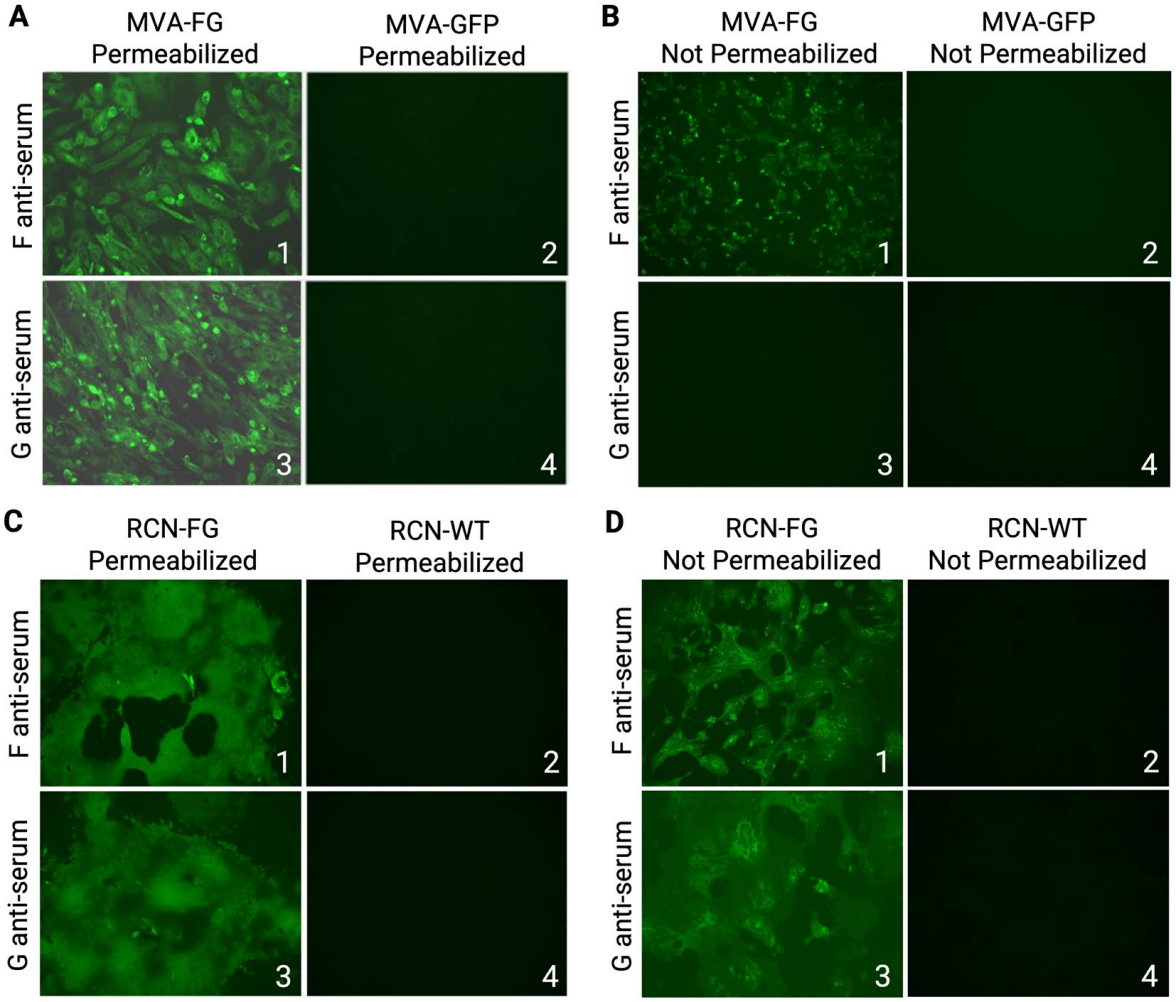
The cellular location of the F and G proteins expressed by recombinant virus vectors MVA-FG and RCN-FG was determined using immunofluorescence. The cell membrane of CEF cells infected with MVA-FG or MVA-GFP and Vero cells infected with RCN-FG or RCN-WT were either permeabilized or left intact. (**A**–**B)** show intracellular and membrane protein localization in CEF cells infected with MVA-FG or MVA-GFP control (**C**–**D)** indicate protein localization in Vero cells infected with RCN-FG or RCN-WT control. Cells were visualized at 10X magnification under fluorescent microscopy (AMG EVOSfl, Thermo Fisher Scientific, Waltham, MA), and images were taken under identical conditions to ensure consistency.

We assessed the expression and cellular localization of the F and G proteins by immunofluorescence microscopy of MVA-FG and RCN-FG-infected cells (Fig. 4). F protein expression was identified in permeabilized (Fig. 4A1) and nonpermeabilized (Fig. 4B1) CEF cells infected with the MVA-FG virus but not in the MVA-GFP-infected control cells. G expression was detected intracellularly in MVA-FG infected cells (Fig. 4A3), but no signal was observed on the cell membranes (Fig. 4B3) or in MVA-GFP-infected cells. However, western blot analysis demonstrated that the G protein is secreted into the supernatant (Supplementary Fig. 2D Lane 3). For the RCN-FG virus, the expression of the F (Fig. 4C1 and D1) and G (Figure 4C3 and D3) proteins is observed intracellularly and on the cell surface; no fluorescence is detected in RCN-WT-infected Vero cells. Western blot analysis (Supplementary Fig. 3) confirms protein expression of the F and G proteins intracellularly and extracellularly for RCN-FG infected cells while RCN-WT infected Vero cells lack these protein bands. Raw western blots for MVA-infected cells and RCN-infected cells can be found in Supplementary Figs. 4 and 5, respectively.

While the RCN-FG vaccine displayed the formation of syncytia and both F and G protein localization on the cell membrane was confirmed via immunofluorescence, the same could not be said for the MVA-FG vaccine. It is hypothesized based on the lack G protein localization via immunofluorescence, lack of syncytia formation in infected cells, and the detection of the G protein in the western blot, that the G protein is being secreted into the supernatant and is not membrane-anchored for the MVA-FG vaccine.

## Post-immunization safety monitoring of mice vaccinated with MVA-FG and RCN-FG

**Figure 5:**
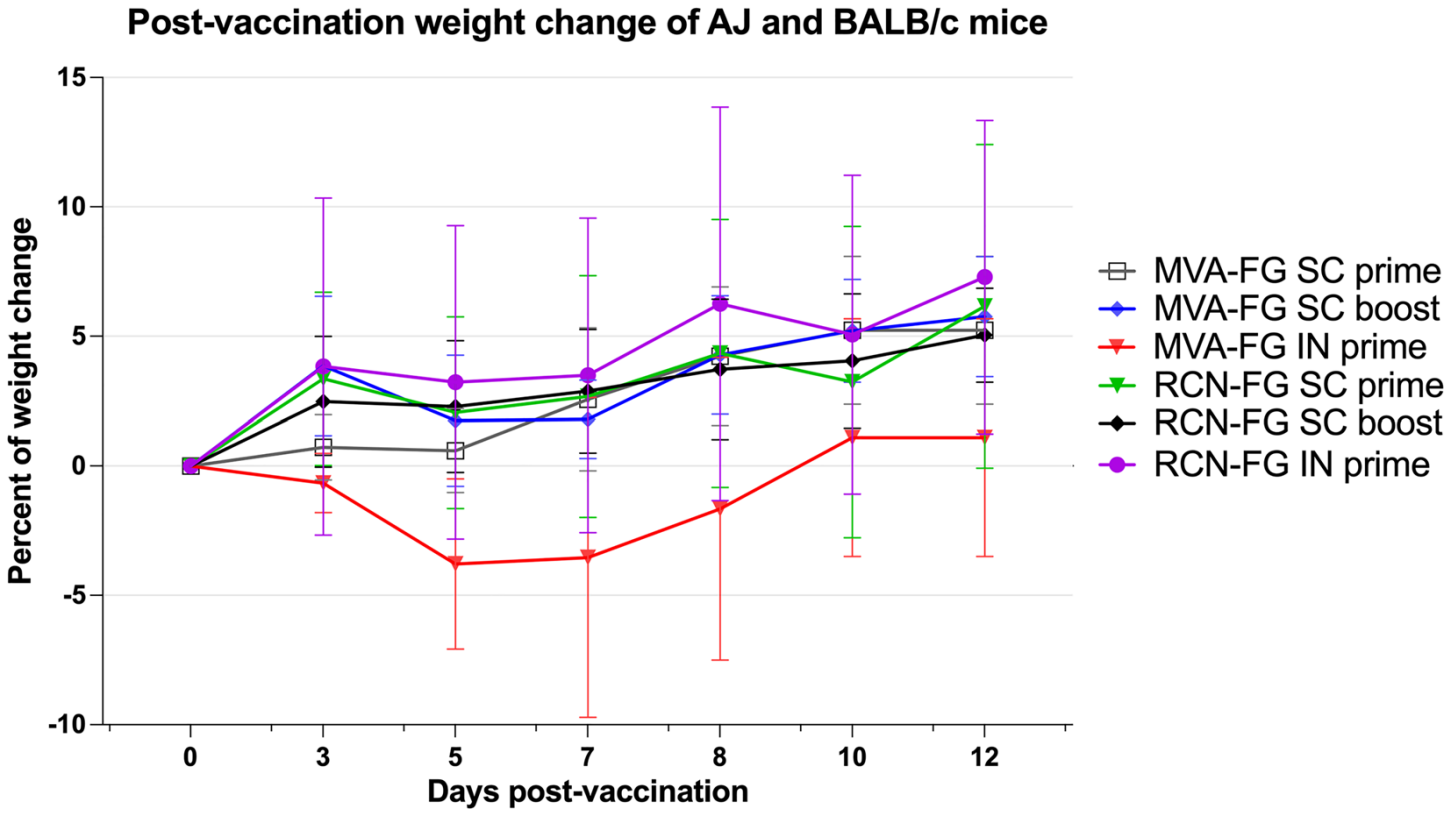
Percentage of weight change (± SD) of BALB/c and AJ mice p.v. BALB/c and AJ mice were monitored daily for two weeks following vaccination. Mice were weighed three times per week using an initial weight taken on the day of the initial vaccination as a baseline for comparison. Weights were expressed as the percentage of weight change from the baseline.

Mice were vaccinated with one or two doses via SC and one dose via IN immunization with 10^8^ PFU of either MVA-FG or RCN-FG vaccine candidates. Animals were weighed three times per week for a period of two weeks and monitored daily for any changes in health status by UW-Madison animal care staff. Mouse body weights post-vaccination (p.v.) were compared to the baseline weight taken immediately prior to vaccination. Interestingly, the only group that showed a decrease in weight were mice immunized IN with MVA-FG (Fig. 5). MVA-FG IN mice lost less than 5% of body weight compared to their baseline weight following vaccination until day 5 p.v. After 5 days p.v, MVA-FG IN mice began increasing in weight until day 10 where they surpassed their initial baseline weight. RCN-FG vaccine groups and MVA-FG groups vaccinated SC had a steady increase in weight p.v. throughout the two-week period. The limited weight loss p.v. and no adverse health events reported by veterinary care staff corroborate previous viral vector safety data and provide initial safety data for these vaccine candidates to undergo further studies for rigorous safety testing.

## NiV-F and NiV-G specific antibody levels induced in mice

**Figure 6:**
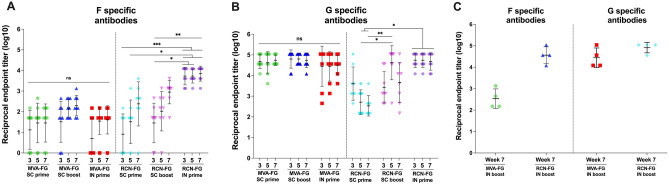
Serum antibody titers in mice vaccinated with 10^8^ PFU of MVA-FG or RCN-FG. Mice received one immunization (prime) with MVA-FG (circle; green) or RCN-FG (diamond; light blue) via SC route, one IN immunization (prime) with MVA-FG (square; red) or RCN-FG (hexagon; violet) vaccine candidates; or two SC immunizations (boost) with MVA-FG (triangle; blue) or RCN-FG (inverted triangle; pink) vaccine candidates 3 weeks apart. Individual data points were taken from each mouse on weeks 3, 5, and 7 post-vaccination and analyzed per group. (**A**–**B)** Anti-F and G IgG antibody titers in serum (geometric mean titer [GMT] ± geometric standard deviation [GSD]) from BALB/c and AJ mice vaccinated with MVA-FG or RCN-FG respectively. Serum samples were collected on weeks 3, 5, and 7 following the initial vaccination. (**C)** Serum antibody titers (GMT ± GSD) in BALB/c and AJ mice that were vaccinated four weeks apart with two doses of either MVA-FG or RCN-FG intranasally. Serum was taken 7 weeks post-prime to assess F and G-specific antibodies. Statistical analysis was performed using a one-way Kruskal Wallis one-way ANOVA, followed by Dunn's multiple comparison test. *p* Values < 0.05 (alpha) were considered statistically significant with *p < 0.05, **p < 0.01, ***p < 0.001, ns = not significant (*p* > 0.05).

Mice were vaccinated with either MVA-FG or RCN-FG viral vectors using one or two doses via SC injection or one dose via IN injection (Fig. 2). The reciprocal endpoint IgG antibody titers were measured using ELISA from serum samples collected on weeks 3, 5, and 7 (Fig. 6). Antibody titers were expressed as the logarithm base 10 geometric mean titer (GMT) per group. In terms of anti-F antibody titers (Fig. 6A), there were no statistical differences between all MVA-vaccinated groups. All three groups generally induced low levels at week 3, which increased when measured at weeks 5 and 7. Specifically, the SC boost group showed higher F titer levels at weeks 5 and 7 than the SC prime and IN groups.

In contrast, RCN-vaccinated groups displayed differences in F antibody titers (Fig. 6A). Overall, the IN group consistently induced higher levels at all three-time points than the other two groups. On the other hand, both SC groups showed an increase in titer levels from week 3 to week 7, but lower than IN groups. Statistical differences at weeks 3 and 5 indicated that the IN group induced higher levels than SC prime and SC boost at week 3. However, there were no differences between IN and SC prime groups at week 7 and between IN and SC boost at weeks 5 and 7. Notably, F titers from animals immunized with RCN-FG IN were higher than levels observed in all three MVA-vaccinated groups (Fig. 6A).

Overall, the G-specific antibody titers induced in mice vaccinated with MVA-FG or RCN-FG (Fig. 6B) were higher than antibody titers seen against the F protein. BALB/c mice immunized with the MVA-FG vaccine showed statistically comparable G antibody titers among all MVA-vaccinated groups but with a higher mean antibody titer seen in groups vaccinated SC versus IN.

High G antibody titers were achieved at all three weeks tested for the IN-treatment group for RCN-FG vaccinated AJ mice. Interestingly, titer levels induced in the SC boost group peaked at week 5 and were similar to the IN group but showed a reduction at week 7 comparable to levels obtained at week 3. In contrast, the SC prime group peaked at week 3 but gradually induced lower levels at weeks 5 and 7. F specific antibody titers for MVA-FG and RCN-FG IN boosted mice (Fig. 6C) saw an increase at 7 weeks post-prime compared to mice receiving a single dose. Antibody titers against the G protein showed comparable levels between mice receiving one or two doses of either the MVA-FG or the RCN-FG vaccines.

In the flow cytometry T cell memory study (Fig. 3), serum was collected from BALB/c and AJ mice vaccinated with two doses of either MVA-FG or RCN-FG IN four weeks apart and the levels of circulating antibodies 7 weeks after prime for IN groups were measured. F antibody titers in MVA-FG and RCN-FG vaccinated mice showed an increase in the mean antibody titer following an IN boost, while G mean antibody titers remained comparable to levels after a single dose for both MVA-FG and RCN-FG-vaccinated mice.

Both vaccines produced high levels of circulating antibodies against both the F and the G glycoproteins and were assessed based on vaccine administration route. Mice vaccinated with MVA-FG did not have antibody titer differences when using different administration routes suggesting route of administration could be variable. Unlike MVA-FG vaccinated mice, RCN-FG vaccinated mice did show differences in antibody production levels based on vaccine administration route, suggesting the IN vaccine delivery route would outperform the SC route in generating circulating antibodies.

## Neutralizing antibodies induced in mice

**Figure 7:**
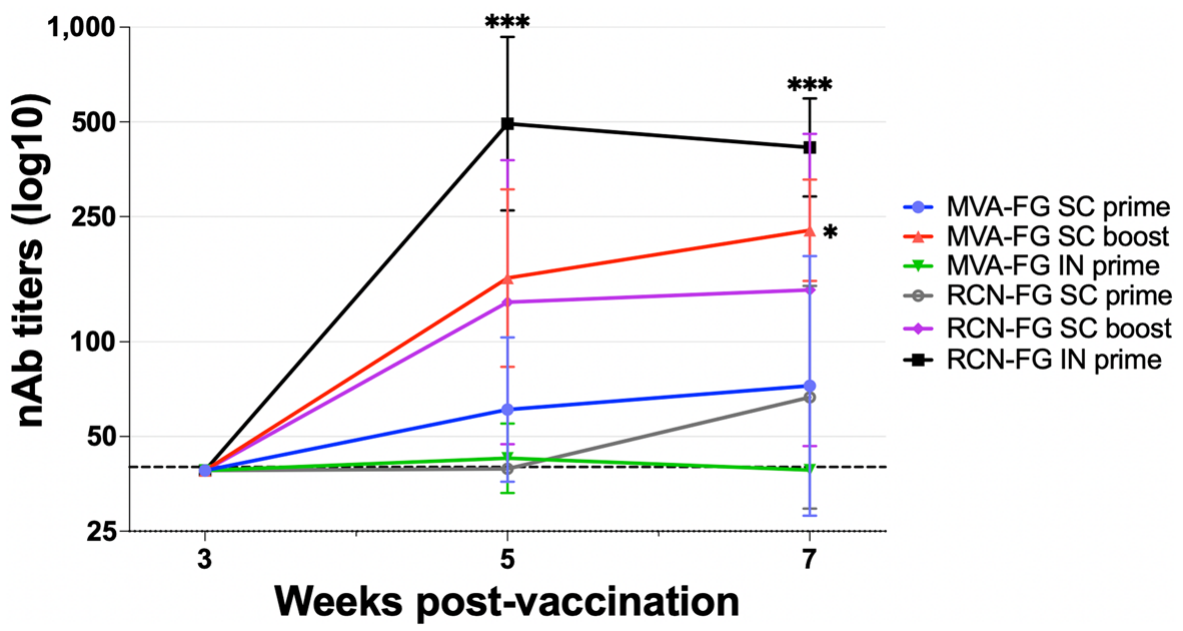
Neutralizing antibody titers against the NiV Bangladesh strain in serum samples collected in weeks 3, 5, and 7 from BALB/c and AJ mice. Animals were primed subcutaneously, boosted subcutaneously, and primed intranasally with MVA-FG or RCN-FG. Samples were run in duplicate, and reciprocal dilutions were considered positive for virus neutralization if both duplicates were negative for CPE 6 days p.i. Data is represented as the GMT of neutralizing antibodies per group of animals. Statistical analysis was performed using a one-way Kruskal Wallis one-way ANOVA, followed by Dunn's multiple comparison test. P-values < 0.05 (alpha) were considered statistically significant with *** p < 0.001, **** p < 0.0001, ns = not significant. The dashed line shows the limit of detection.

Mouse serum collected on weeks 3, 5, and 7 was evaluated for the presence of nAbs against the NiV Bangladesh strain (Fig. 7). Serum was tested in duplicate for 100% neutralization via CPE visualization 6 days p.i. Reciprocal dilution titers were considered neutralizing at the lowest dilution in which both duplicates showed no CPE. Although none of the vaccine candidates induced nAbs at week 3 after the first immunization, in the MVA-FG vaccinated animals, the SC boost group induced the highest mean titer of nAbs, which increased from week 5 (1:159.5) to week 7 (1:226.3). The MVA-FG SC prime reached a final titer on week 7 (1:72.4), while IN group peaked at week 5 (1:42.7). RCN-vaccinated mice had the highest overall titer of nAbs in the IN prime group on weeks 5 (1:495) and 7 (1:419), followed by the RCN-FG SC boost group, which reached 133.7 in week 5 and 146.3 in week 7, and RCN-FG SC prime which reached a final titer of 66.6 on week 7.

Overall, both vaccines were able to induce neutralizing antibodies. For mice vaccinated with MVA-FG, the level of neutralizing antibodies produced was dependent on vaccine administration route with the SC groups having significantly higher neutralizing antibodies the IN group. Furthermore, mice vaccinated SC also showed an increase in the level of neutralizing antibodies generated with two doses versus a single dose. Therefore, while circulating antibodies were unaffected by vaccine administration route, the ability of the antibodies to neutralize the virus was affected. Mice vaccinated with RCN-FG had differences like the overall level of circulating antibodies seen in serum. The RCN-FG IN group had significantly high levels of neutralizing antibodies generated quickly compared to SC administration, affirming the IN group as the better vaccine administration route.

## IFN-γ, IL-2, and TNF-α secreting cells stimulated by NiV-F and G glycoproteins

**Figure 8:**
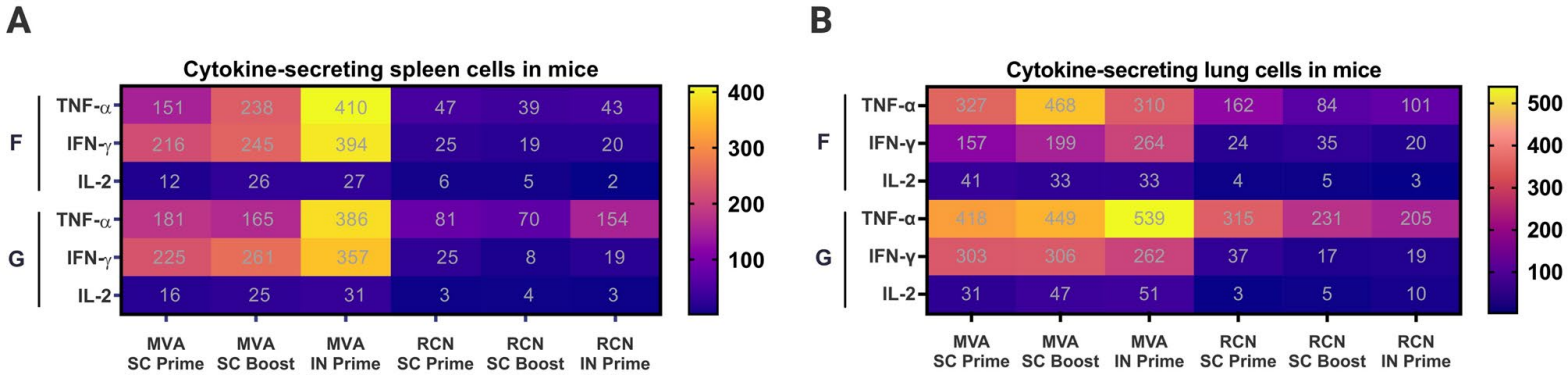
Cytokine secreting cells from lung and spleen cells in BALB/c and AJ mice vaccinated with MVA-FG and RCN-FG. (**A)** Cytokine-secreting spleen cells in mice vaccinated with MVA-FG and RCN-FG. (**B)** Cytokine-secreting lung cells in mice vaccinated with MVA-FG and RCN-FG. Animals were primed subcutaneously, boosted subcutaneously, and primed intranasally. Tissues were collected 7 weeks after the initial vaccination and were stimulated with the full-sized purified F and G proteins. The level of cytokine-secreting cells was expressed as the number of secreting cells per 300,000 cells.

Splenocytes (Fig. 8A) and lung cells (Fig. 8B) collected from BALB/c and AJ mice 3 weeks post-boost or 7 weeks post-prime were analyzed for IFN-γ, IL-2, and TNF-α cytokine secreting cells in response to stimulation by the full-sized NiV-F and G glycoproteins. The MVA-FG SC and IN groups showed high levels of all three cytokines. Based on vaccine administration route, MVA-FG IN groups had higher levels of TNF-⍺, IFN-γ, and IL-2-secreting cells in splenocytes compared with groups receiving MVA-FG SC for both the F and the G antigen. SC primed and boosted groups showed similar levels of TNF-⍺, IFN-γ, and IL-2 cytokine-secreting cells for both cell types when stimulated with the F and G antigens.

RCN-FG vaccinated mice had low levels of TNF-⍺, IFN-γ, and IL-2 cytokine-secreting cells in both splenocytes and lung cells against both the F and G antigen. However, a higher percentage of TNF-⍺ secreting lung cells was detected against the G antigen compared with splenocytes for mice vaccinated SC. For mice vaccinated IN, similar percentages of TNF-⍺ secreting cells were detected in both splenocyte and lung cells against the G antigen.

Cytokine-secreting cells in mice vaccinated with MVA-FG had the highest level of expression via IN vaccine route, with SC groups showing no significant difference between SC groups that received one or two doses. RCN-FG vaccinated mice did not show significant differences between vaccine administration groups, however a higher percentage of TNF-⍺ secreting cells was noted in splenocytes for mice vaccinated IN.

### T cell memory assessed via flow cytometry

**Figure 9:**
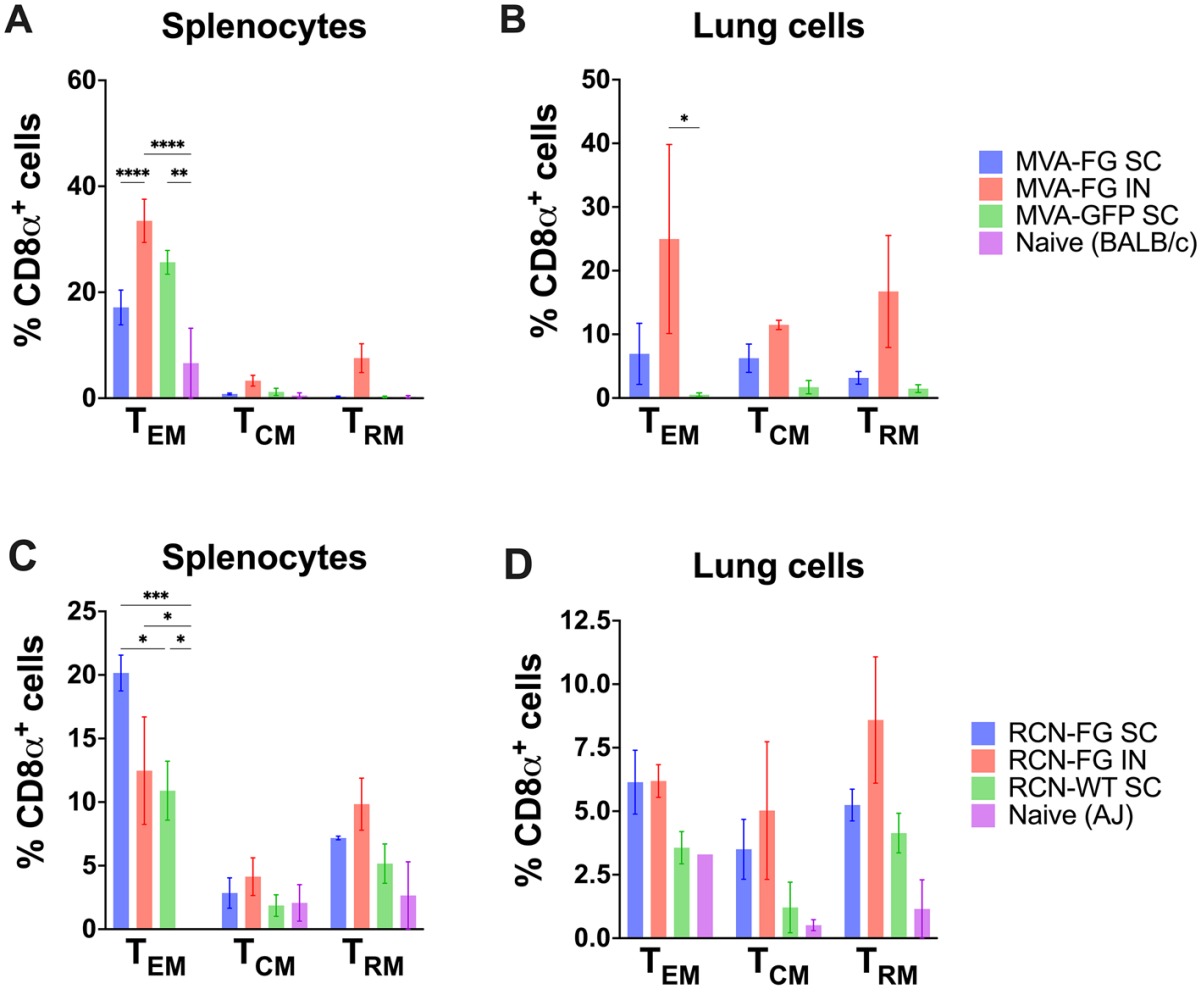
CD8⍺^+^ effector, central, and tissue-resident memory T cells. Phenotypic expression of CD8⍺^+^ effector (CD62L^+^ CD44^-^), central (CD62L^+^ CD44^+^), and tissue-resident (CD103^+^ CD69^+^) memory T cells was evaluated in BALB/c and AJ mice 5 weeks post-boost. (**A)** Splenocytes stimulated with F and G peptides from naive BALB/c and MVA-vaccinated mice. (**B)** Lung cells stimulated with F and G peptides from naive BALB/c and MVA-vaccinated mice. (**C)** Splenocytes stimulated with F and G peptides from naive AJ and RCN-vaccinated mice. (**D)** Lung cells stimulated with F and G peptides from naive AJ and RCN-vaccinated mice. Data were represented with mean ± standard error of the mean (SEM) with n = 1–4 for vaccination groups and n = 1 for naive mice. Samples were excluded based on poor cell recovery during tissue harvesting. Statistical analysis was conducted using a one-way ANOVA and a Tukey’s multiple comparison test after the discovery of significant differences.

BALB/c and AJ mice were vaccinated with the MVA-FG or the RCN-FG vaccine SC and IN in two-dose schedule 4 weeks apart. Additional groups received an MVA-GFP or an RCN-WT virus SC at vector controls. One naive mouse from each species was kept as naive control. Five weeks post-boost, mouse splenocytes, and lungs were collected from BALB/c and AJ mice and analyzed for effector (T_EM_), central (T_CM_), and resident tissue (T_RM_) CD4^+^ (Supplementary Fig. 6) and CD8⍺^+^ T cells (Fig. 9).

Splenocytes from MVA-vaccinated BALB/c mice (Fig. 9A) had significant effector memory expression in MVA-FG IN groups compared to MVA-FG SC (*p* < 0.0001), and naive mice (*p* < 0.0001). Mice vaccinated with MVA-GFP SC also showed significant expression of effector memory compared to naive mice (*p* = 0.0053). For central and resident memory CD8⍺^+^ T cells, no significant differences were found, however, MVA-FG IN mice had the highest level of expression compared to MVA-FG SC, MVA-GFP SC, and naive mice. Lung cells (Fig. 9B) were collected from BALB/c mice vaccinated with MVA-FG SC and IN and MVA-GFP SC with low cell collection in naive mice being excluded from the data set. MVA-FG IN mice had the highest expression of all memory CD8⍺^+^ T cell subsets in the MVA-FG IN group, significantly greater than the MVA control SC group for effector memory expression (*p* = 0.0455). MVA-FG SC groups also saw some effector, central, and tissue resident CD8⍺^+^ T cell expression, with little to no expression seen in MVA-GFP SC groups.

Splenocytes collected from AJ mice (Fig. 9C) had significantly high expression of T_EM_ in RCN-FG SC mice compared to RCN-WT SC (*p* = 0.0264) and naive AJ mice (*p* = 0.0002). Mice vaccinated with RCN-FG IN (*p* = 0.0205) and RCN-WT SC (*p* = 0.0470) also showed significant effector memory CD8⍺^+^ expression compared with naive mice. While no significant differences were seen for central or tissue resident memory CD8⍺^+^ splenocytes, RCN-FG SC and IN mice had the higher expression of both phenotypes compared to RCN-WT SC vaccinated mice and naive AJ mice. For lung cells (Fig. 9D) from AJ mice, no significant differences were seen between groups for CD8⍺^+^ T cell memory subsets, however higher levels of expression was seen in mice vaccinated with RCN-FG SC and IN.

Importantly, both vaccines generated CD8ɑ^+^ T_EM_, T_CM,_ and T_RM_ responses in lung and spleen cells from vaccinated mice upon stimulation from peptides from the F and G proteins. While the MVA-FG vaccine generated the highest level of circulating antibodies when given SC, the highest phenotypic expression of effector, central, and tissue resident CD8ɑ^+^ T cells in both splenocytes and lung cells was detected when given intranasally which should be explored in future studies. Excitingly, the RCN-FG vaccine candidate generated effector and tissue resident CD8ɑ^+^ T cells via IN and SC administration in both splenocytes and lung cells supporting oral vaccination as a good means of immune stimulation using this vaccine.

### Mucosal antibodies assessed via ELISA

**Figure 10:**
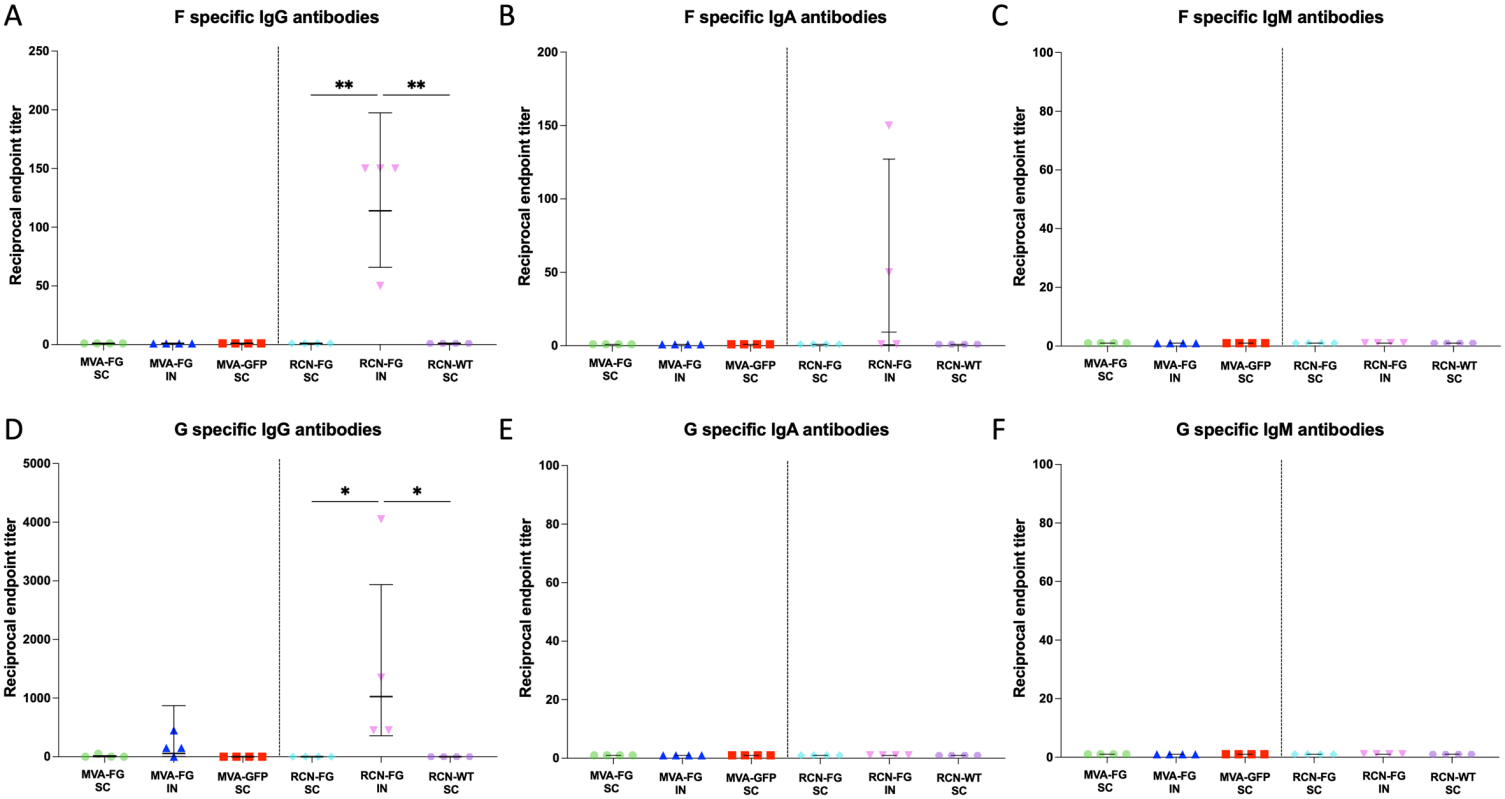
The level of F and G-specific IgG, IgA, and IgM antibodies in BALF from BALB/c and AJ mice. Mice were given two doses of MVA-FG SC and IN, MVA-GFP SC, RCN-FG SC and IN, or RCN-WT four weeks apart, and BALF was collected five weeks post-boost. Antibody titers were displayed per animal while the GMT ± GSD was displayed per group. F specific (**A)** IgG, (**B)** IgA and (**C)** IgM antibody titers. G specific (**A)** IgG, (**B)** IgA and (**C)** IgM antibody titers. Statistical analysis was performed using a one-way Kruskal Wallis one-way ANOVA, followed by Dunn's multiple comparison test. *p* Values < 0.05 (alpha) were considered statistically significant with *p < 0.05, **p < 0.01, ***p < 0.001, ns = not significant (*p* > 0.05).

BALF was collected from BALB/c and AJ mice 5 weeks post-boost, and the F and G antibody titers were assessed for IgG, IgA, and IgM antibody types to see if antibodies were generated in the respiratory tract along the natural route of infection. F-specific IgG antibodies (Fig. 10A) were detected in the RCN-FG IN group at a GMT of 125 which was significantly higher than the RCN-FG SC (*p* = 0.0032) and the RCN-WT SC (*p* = 0.0032) groups which had no detectable antibodies. For F-specific IgA (Fig. 10B), the RCN-FG IN group was the only group that had any detectable IgA antibody titers with a geometric mean of 50. For F-specific IgM (Fig. 10C), no antibodies against the F protein were detected in vaccine or vector control groups. IgG antibody titers against the G protein (Fig. 10D) had a GMT of 13.25 in MVA-FG SC groups, and 187.8 in MVA-FG IN groups, with no detectable antibodies in the MVA vector control group. For AJ mice, IgG antibody titers had a GMT of 1575 in the RCN-FG IN group which was significantly higher than the RCN-FG SC (*p* = 0.0155) and RCN-WT SC group (*p* = 0.0155). No IgA (Fig. 10E) or IgM (Fig. 10F) G-specific antibodies were detected for any vaccine or vector control groups in AJ mice. As hypothesized, neither vaccine produced mucosal antibodies when vaccinated subcutaneously with one or two doses, however, while neither vaccine produced F or G-specific IgM antibodies, both vaccines had detected IgG antibody titers for intranasally vaccinated mice. Notably, high G-specific antibody titers were seen for both vaccines and the generation of F-specific IgG and IgA antibodies were detected for mice vaccinated with RCN-FG. Interestingly, while F-specific circulating antibodies were produced, no F-specific antibodies were detected in respiratory tract of MVA-FG IN groups.

## Discussion

While vaccine candidates against NiV are in development, none are commercially available for this fatal disease. This study assessed the immunogenicity and safety of poxvirus-based vaccines against NiV. The MVA-FG and RCN-FG vaccines induced high levels of anti-F and G circulating antibodies through subcutaneous and intranasal administration. Furthermore, BALF collected from vaccinated mice had detectable F-specific IgG and IgA antibodies in the RCN-FG IN group and G-specific IgG antibodies in the MVA-FG and RCN-FG IN groups. Most importantly, each vaccine generated high neutralization activity against the NiV Bangladesh (NiV_B_) strain. A single dose of the RCN-FG vaccine candidate, given intranasally, successfully neutralized 100% of NiV_B_ at a mean dilution of 1:495, while the MVA-FG vaccine given via boosted SC route neutralized 100% of NiV_B_ at a dilution of 1:240. While challenge studies were not conducted in this study, studies have shown that nAb levels are predictive of immune protection in various passive immunization studies in NiV animal challenge models^15,42^; however, challenge studies will need to be conducted to assess protection and efficacy.

Regarding the cellular immune response, splenocytes and lung cells harvested from mice vaccinated with MVA-FG and RCN-FG vaccine candidates secreted TNF-⍺, IFN-γ, and IL-2 in response to F and G protein stimulation. MVA-vaccinated mice had higher levels of cytokine expression compared to RCN-vaccinated mice. MVA-FG mice vaccinated IN had the highest expression of TNF-⍺, IFN-γ, and IL-2 in splenocytes. Furthermore, CD4^+^ and CD8ɑ^+^ T cell memory responses showed the generation of T_EM_, T_CM,_ and T_RM_ in both MVA-FG and RCN-FG vaccinated mice. Specifically, the MVA-FG vaccine given intranasally saw the highest phenotypic expression of effector, central, and tissue resident CD8ɑ^+^ T cells in both splenocytes and lung cells compared to vector control and naive mice. The RCN-FG vaccine candidate generated high effector memory CD8ɑ^+^ T cells when given SC and produced tissue resident CD8ɑ^+^ T cells via IN administration in splenocytes. In lung cells, the RCN-FG vaccine showed higher effector, central, and tissue resident CD8ɑ^+^ T cell expression compared with RCN-WT SC and naive mice. Again, while challenge studies would need to be conducted to assess protection, cytokine secretion and the generation of T_EM_, T_CM_, and T_RM_ in both spleen and lung cells are comparable to levels seen in swine challenge studies that confer protection^43^.

Recombinant poxvirus vectors are easily constructed and provide high immunogenicity and enhance the induction of cellular and humoral-mediated immune responses^44^. Furthermore, unlike some viral vectors, poxviruses do not carry the risk of DNA recombination in target cell genomes due to the characteristic of cytoplasmic replication. While the consideration of vector immunity is an important area of focus, studies have shown that a single recombinant poxvirus can successfully express transgenes after multiple immunizations^45^. It is hypothesized that the antibodies produced against poxvirus target the virion during release from infected cells and not the entry/attachment phase at initial infection^45^. With this is mind, people recently infected with poxvirus would generate antibodies that prevent cell-to-cell spread of the viral vectors at the entry/attachment phase^45^. With one of the last known cases of smallpox in Bangladesh and the deployment of the MVA vaccine to counteract recent monkeypox infections in humans, long-term vector immunity should be studied in greater depth^46^. In addition, the poxvirus viral vectors used as the delivery platform for the NiV F and G proteins are safe and effective^18–21,23–29^. In wildlife, both the MVA and RCN viral vectors are safe and effective in bat and pig populations^47,48^. In this study, mice vaccinated with the MVA-FG and RCN-FG vaccines saw limited weight loss post-vaccination suggesting an initial safe vaccine profile. However, further preliminary data would need to be collected to determine the final safety profile prior to clinical trials or animal challenge studies such as cytokine production, and monitoring for immediate or long-term adverse events.

Additionally, the MVA and RCN viral vectors elicit high humoral immune responses in bats through oral immunization necessary for practical, wide-scale animal vaccinations in the field^49^. The RCN vectored vaccine presents a promising wildlife vaccine against NiV in both the bat reservoir and pigs, an amplifying host of NiV, to reduce the spread from wildlife to human populations and prevent the economic disruption caused by swine exportation to areas without the competent host reservoir. By being able to vaccinate both humans and wildlife, the MVA vaccine candidate presents a possible One-Health vaccine candidate and creates an avenue for disease eradication.

Current vaccine approaches for Nipah rely on subunit, mRNA, VLP, DNA, or viral vector strategies^48–57^. Currently, an Equivac^®^ HeV recombinant subunit vaccine, licensed for horses in Australia against Hendra virus and NiV, is undergoing phase I clinical trials for humans^48,58^. Like the vaccines proposed in this study, the Equivac® HeV vaccine focuses on a One-Health approach against Hendra virus by targeting the transmission from horses to humans^59^. While this vaccine has proven to protect both ferrets and African green monkeys from fatal NiV challenges, only partial protection was obtained in pigs due to their requirement of both cellular and humoral immune responses for complete protection^60^. Recombinant vectored vaccines have many promising candidates in development tailored toward both humans and pigs including vaccines such as the recombinant rabies virus (NIPARAB), a vesicular stomatitis virus (rVSV-ΔG-NiV_B_G), canarypox virus (ALVAC-F/G) vaccines, and ChAdOx1 NiV_B_, a simian adenovirus vectored vaccine^53,61–63^. Although promising, vaccine candidates against NiV fail to include the reservoir host as a vaccine target, but instead focus on the transmission between humans and swine. Due to the infrequent nature of NiV outbreaks and limited wide-scale marketability, vaccination campaigns may focus on rapid vaccine deployment in outbreak regions^64^. By deploying a vaccine capable of use in the reservoir host, wildlife vaccination can focus on preventing the spread of NiV between migratory bat regions and to other susceptible species such as pigs and humans during seasonal outbreaks. While vaccine models have been able to induce protective immune responses, few vaccine candidates (RABV and VSV) have tested protective efficacy via intranasal administration, significantly reducing their practical application in animal populations that require mucosal delivery. For current vaccine models to fully target the transmission cycle of NiV, they would need to entail a collaborative effort to halt transmission between the reservoir host, the amplifying host, and humans. Without a One Health-based vaccine approach capable of targeting the transmission cycle of NiV, control and prevention focus on mitigation after an outbreak occurs. Since NiV persists in bat reservoirs, this reservoir must be targeted through vaccine strategies to prevent wide-scale outbreaks.

These results present two promising poxvirus vaccine candidates against NiV that may provide an avenue for protection in both humans and animals involved in NiV transmission. The stimulation of high humoral immune responses through both subcutaneous and intranasal routes highlights their potential as wildlife vaccines in humans, swine, and the reservoir bat species. Finally, the induction of CD8^+^ T cell responses emphasizes the induction of cellular immune responses required for complete protection against NiV in swine species.

## Supplementary Information

Supplementary Information.

## Data Availability

All data are available within the manuscript, supplementary material, or at the corresponding author’s request. MVA-FG (https://www.ncbi.nlm.nih.gov/nuccore/OQ352141) and RCN-FG (https://www.ncbi.nlm.nih.gov/nuccore/OQ352142) DNA construct sequences are available online through GenBank (Accession OQ352141.2 and OQ352142.2, respectively). Information regarding the NiV Bangladesh strain (Accession MK673564.1) used for virus neutralization assays is available through GenBank (https://www.ncbi.nlm.nih.gov/nuccore/1802790317).
