# Supplementary material for: Immunogenicity of poxvirus-based vaccines against Nipah virus
Source: Sci Rep. 2023 Jul 14;13:11384. doi: 10.1038/s41598-023-38010-2 (PMC10349127; doi:10.1038/s41598-023-38010-2)
Supplement: Supplementary file 1 — Supplementary Information. [file 41598_2023_38010_MOESM1_ESM.docx]

**SUPPLEMENTARY DATA**

*Supplementary Materials and Methods
Western Blot Analysis*

CEF cells were infected with MVA-FG and MVA-GFP, while Vero cells were infected with RCN-FG and RCN-WT diluted in OptiMEM (Thermo Fisher Scientific, Waltham, MA, Cat. No.: 22600134) at an MOI = 10 PFU/cell. Forty-eight hours p.i, the supernatant was collected and filtered using Nanostep 30K Omega filters (Pall Corporation, NY, USA, Cat. No.: MCP030C41) and resuspended in 4X Laemmli Sample Buffer (Bio-Rad, Richmond, CA, Cat. No.: 1610747) containing 5% 2-mercaptoethanol (Sigma Aldrich, Germany, Cat. No.: M6250). Infected cells were scraped, resuspended in 1X PBS, and recovered by centrifugation at 11,200 g at RT for 5 min. The cell pellet was washed with PBS, centrifuged at 11,200 g at RT for 5 min, and resuspended in 4X Laemmli Sample Buffer (Bio-Rad, Richmond, CA, Cat. No.: 1610747) containing 5% 2-mercaptoethanol (Sigma Aldrich, Germany, Cat. No.: M6250). NiV fusion protein (gF) exodomain (Native Antigen, Kidlington, Oxford, UK, SKU: REC31632) and NiV glycoprotein G (gG) exodomain (Native Antigen, Kidlington, Oxford, UK, SKU: REC31637) were used as positive controls. Samples were heated to 95°C for 5 min. SDS-PAGE resolved samples were then transferred to a nitrocellulose membrane (Bio-Rad, Richmond, CA, Cat. No.: 1704158). The membrane was blocked overnight at 4°C in 5% milk diluted in TBS-T. Mouse protein-specific antiserum produced in-house was diluted in blocking buffer and 5% FBS (1:500 dilution) and incubated overnight at 4°C. Membranes were washed with TBS-T and incubated with goat anti-mouse IgG (H + L) peroxidase conjugate (Thermo Fisher Scientific, Waltham, MA, Cat. No.: 31430) diluted in blocking buffer and 5% goat serum (1:3,000 dilution) for 1 hr. The membranes were then washed with TBS-T, and Pierce 1-step Ultra TMB Blotting solution (Thermo Fisher Scientific, Waltham, MA, Cat. No.: 37574) was added for protein visualization. Membranes were scanned with an Epson Scanner (EPSON Perfection 4490 Photo) using the Epson Scan Utility v3.24 software.

*Supplementary Results*

**Supplementary Table 1.** Antibodies and dilutions used in the flow cytometry memory T cell studies.

| **Antibody** | **Clone** | **Dilution** | **Manufacturer, Catalog number** |
| --- | --- | --- | --- |
| Brillant ultraviolet 395 Rat Anti-mouse CD8⍺ | 53-6.7 | 1:200 | BD, 563786 |
| FITC anti-mouse CD4 | GK1.5 | 1:200 | Biolegend, 100405 |
| PE-Cy7 CD69 | H1.2F3 | 1:200 | Biolegend, 104512 |
| PE-CF594 Rat anti-mouse CD44 | IM7 | 1:200 | BD, 562464 |
| BV711 Rat Anti-mouse CD62L | MEL-14 | 1:200 | BD, 740660 |
| APC anti-mouse CD103 | 2E7 | 1:300 | Biolegend, 121414 |

Western blot analysis for both the infected cell supernatant and cell pellets was performed to ensure both the F and the G proteins were expressed. The expected sizes of NiV’s outer membrane proteins were determined to be 72-75 kDa for G protein depending on glycosylation, the uncleaved form of F (F₀) is 61 kDa, cleaved forms of F are 49 kDa for F₁ and 19 kDa for F₂ [66]. The positive controls received from Native Antigen had an expected molecular weight of ~108 kDa for G protein and ~90 kDa for F protein. Western blot was positive for the cleaved F₁ protein in cells pellets and supernatant infected MVA-FG (Supplementary Figure 2B and 2D) and RCN-FG (Supplementary Figure 3B and 3D) that showed bands at just above 50 kDa. In contrast, cells infected with MVA-GFP and RCN-WT and uninfected cells showed an absence of this band. Both constructs were negative for detecting the uncleaved form of the F protein, F₀, and the cleaved F₂ portion. For NiV-G, western blot analysis detected a band just below 75 kDa for CEF cells and supernatant from cells infected with MVA-FG that was absent in lanes containing supernatant and cell pellets from uninfected CEF cells and CEF cells infected with MVA-GFP (Supplementary Figure 2A and 2C). For Vero cells infected with RCN-FG, a band was detected around 50 kDa in both cells and supernatant that was absent in uninfected Vero cells and infected with RCN-WT (Supplementary Figure 3A and 3C). This lower molecular weight is consistent with other western blot analyses conducted [67] and is believed to be due to Vero cells failing to glycosylate the G protein.


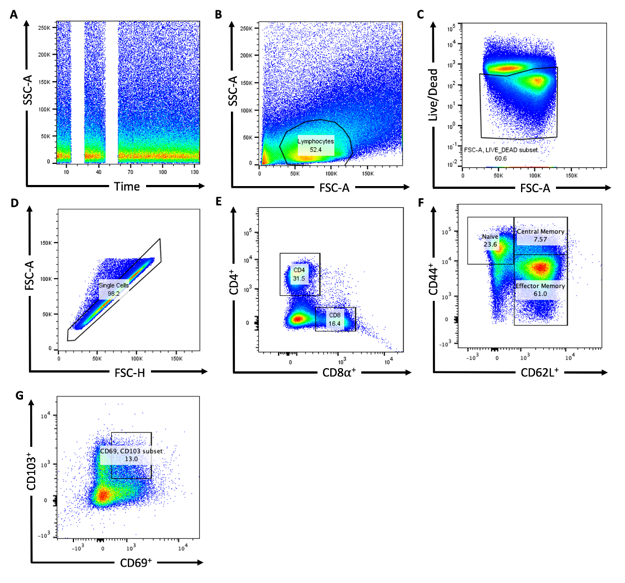


**Supplementary Figure 1.** The gating strategy for flow cytometry analysis. **A)** FlowClean FlowJo software was used to detect and exclude cellular anomalies over time. **B)** Gating strategies selected for proliferating CD4^+^ and CD8α^+^ T cells using a forward (FSC) and side scatter (SSC) dot plot, **C)** dead cells and debris were excluded using a cell viability stain (Aqua Dye), and **D)** a doublet exclusion via forward scatter height (FCS-H) and forward scatter area (FCS-A) dot plot. **E)** Live, CD4^+^ and CD8α^+^ lymphocytes were then gated positive at the FITC axis and the BUV395 axis, respectively, in a FITC-BUV395 dot plot. **F)** CD4^+^ and CD8⍺^+^ effector (CD62L^+^ CD44^-^) and central (CD62L^+^ CD44^+^) memory T cells were gated in a BV711-PE-CF594 dot plot, respectively. **G)** CD4^+^ and CD8⍺^+^ tissue-resident (CD69^+^ CD103^+^) memory T cells were gated positive at the PE-Cy7 axis and the APC axis, respectively, in a PE-Cy7-APC dot plot.


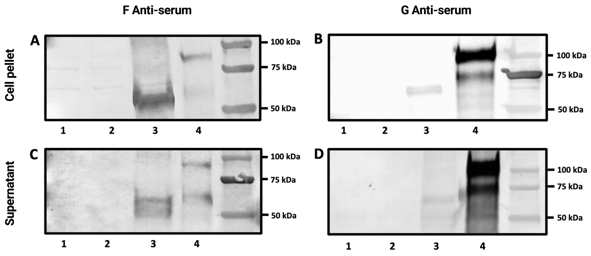


**Supplementary Figure 2.** Western blot images for F and G proteins from the cell pellet and supernatant of CEF cells. Images were cropped and converted to greyscale. Unedited raw images can be found in Supplementary Figure 4. **A)** Protein bands detected in CEF cell pellets using a fusion anti-serum. Lane 1: Uninfected CEF cells. Lane 2: CEF cells infected with MVA-GFP. Lane 3: CEF cells infected with MVA-FG. Lane 4: Purified NiV fusion protein (gF) exodomain (Native Antigen, Kidlington, Oxford, UK, SKU: REC31632). **B)** Protein bands detected in CEF cell pellets using a glycoprotein anti-serum. Lane 1: Uninfected CEF cells. Lane 2: CEF cells infected with MVA-GFP. Lane 3: CEF cells infected with MVA-FG. Lane 4: Purified NiV glycoprotein G (gG) exodomain (Native Antigen, Kidlington, Oxford, UK, SKU: REC31637). **C)** Protein bands detected in the supernatant of CEF cells using a fusion anti-serum. Lane 1: Supernatant from uninfected CEF cells. Lane 2: Supernatant from CEF cells infected with MVA-GFP. Lane 3: Supernatant from CEF cells infected with MVA-FG. Lane 4: Purified NiV fusion protein (gF) exodomain (Native Antigen, Kidlington, Oxford, UK, SKU: REC31632). **D)** Protein bands detected in the supernatant of CEF cells using a glycoprotein anti-serum. Lane 1: Supernatant from uninfected CEF cells. Lane 2: Supernatant from CEF cells infected with MVA-GFP. Lane 3: Supernatant from CEF cells infected with MVA-FG. Lane 4: Purified NiV glycoprotein G (gG) exodomain (Native Antigen, Kidlington, Oxford, UK, SKU: REC31637).

**
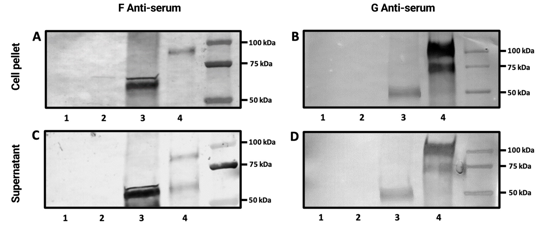
**

**Supplementary Figure 3.** Western blot images for F and G proteins from the cell pellet and supernatant of Vero cells. Images were cropped and converted to greyscale. Unedited raw images can be found in Supplementary Figure 5. **A)** Protein bands detected in Vero cell pellets using a fusion anti-serum. Lane 1: Uninfected Vero cells. Lane 2: Vero cells infected with RCN-WT. Lane 3: Vero cells infected with RCN-FG. Lane 4: Purified NiV fusion protein (gF) exodomain (Native Antigen, Kidlington, Oxford, UK, SKU: REC31632). **B)** Protein bands detected in Vero cell pellets using a glycoprotein anti-serum. Lane 1: Uninfected Vero cells. Lane 2: Vero cells infected with RCN-WT. Lane 3: Vero cells infected with RCN-FG. Lane 4: Purified NiV glycoprotein G (gG) exodomain (Native Antigen, Kidlington, Oxford, UK, SKU: REC31637). **C)** Protein bands detected in the supernatant of Vero cells using a fusion anti-serum. Lane 1: Supernatant from uninfected Vero cells. Lane 2: Supernatant from Vero cells infected with RCN-WT. Lane 3: Supernatant from Vero cells infected with RCN-FG. Lane 4: Purified NiV fusion protein (gF) exodomain (Native Antigen, Kidlington, Oxford, UK, SKU: REC31632). **D)** Protein bands detected in the supernatant of Vero cells using a glycoprotein anti-serum. Lane 1: Supernatant from uninfected Vero cells. Lane 2: Supernatant from Vero cells infected with RCN-WT. Lane 3: Supernatant from Vero cells infected with RCN-FG. Lane 4: Purified NiV glycoprotein G (gG) exodomain (Native Antigen, Kidlington, Oxford, UK, SKU: REC31637).


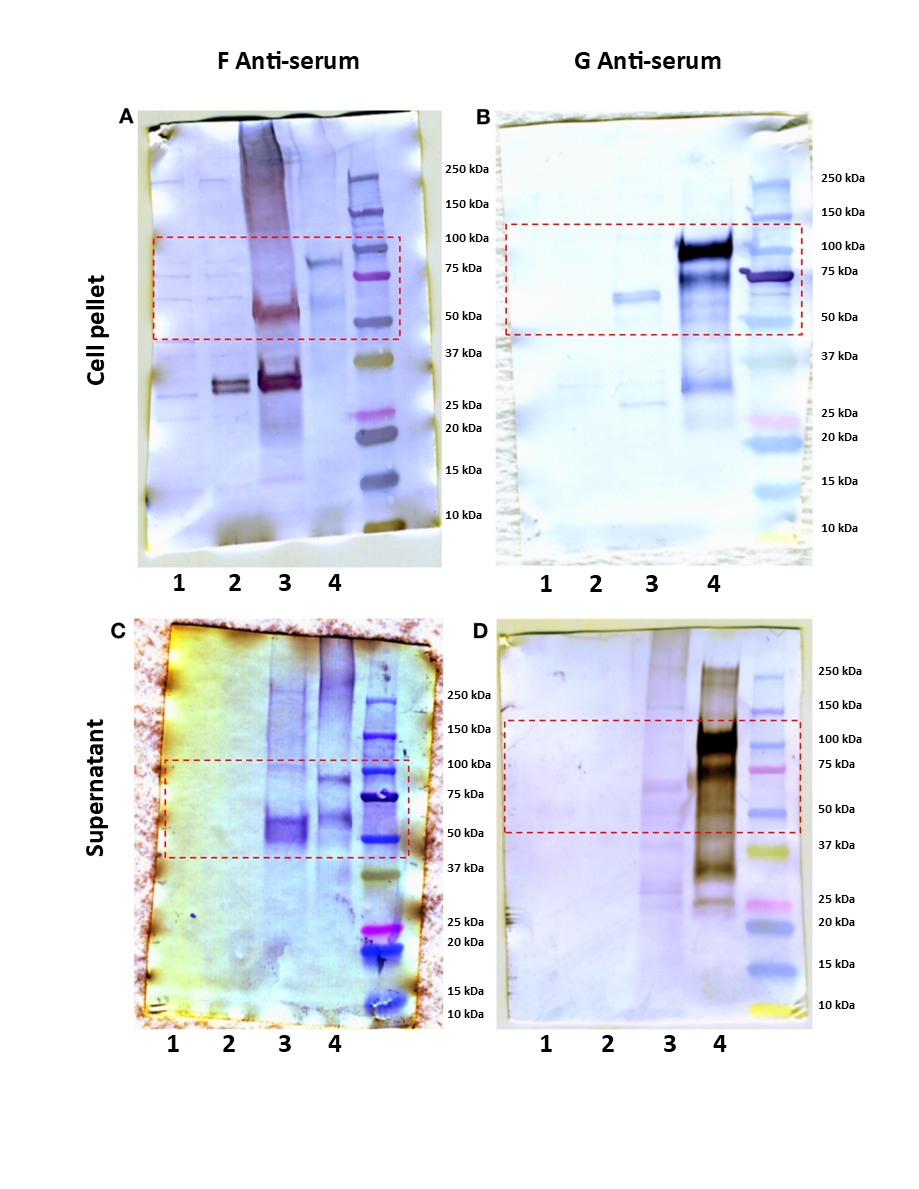


**Supplementary Figure 4.** Raw data for Supplementary Figure 2. Raw western blot images of cell pellet and supernatant samples taken from cells infected with MVA-FG. Western blots were cut prior to antibody hybridization to minimize blank space. Images A-D were cropped along the red-dotted boxes and converted to greyscale as seen in Supplementary Figure 2. **A)** Protein bands detected in CEF cell pellets using a fusion anti-serum. Red boxes outline the section cropped and displayed in Supplementary Figure 2A. Lane 1: Uninfected CEF cells. Lane 2: CEF cells infected with MVA-GFP. Lane 3: CEF cells infected with MVA-FG. Lane 4: Purified NiV fusion protein (gF) exodomain (Native Antigen, Kidlington, Oxford, UK, SKU: REC31632). **B)** Protein bands detected in CEF cell pellets using a glycoprotein anti-serum. Red boxes outline the section cropped and displayed in Supplementary Figure 2B. Lane 1: Uninfected CEF cells. Lane 2: CEF cells infected with MVA-GFP. Lane 3: CEF cells infected with MVA-FG. Lane 4: Purified NiV fusion protein (gF) exodomain (Native Antigen, Kidlington, Oxford, UK, SKU: REC31632). **C)** Protein bands detected in the supernatant of CEF cells using a fusion anti-serum. Red boxes outline the section cropped and displayed in Supplementary Figure 2C. Lane 1: Uninfected CEF cells. Lane 2: CEF cells infected with MVA-GFP. Lane 3: CEF cells infected with MVA-FG. Lane 4: Purified NiV fusion protein (gF) exodomain (Native Antigen, Kidlington, Oxford, UK, SKU: REC31632). **D)** Protein bands detected in the supernatant of CEF cells using a glycoprotein anti-serum. Red boxes outline the section cropped and displayed in Supplementary Figure 2D. Lane 1: Uninfected CEF cells. Lane 2: CEF cells infected with MVA-GFP. Lane 3: CEF cells infected with MVA-FG. Lane 4: Purified NiV fusion protein (gF) exodomain (Native Antigen, Kidlington, Oxford, UK, SKU: REC31632).


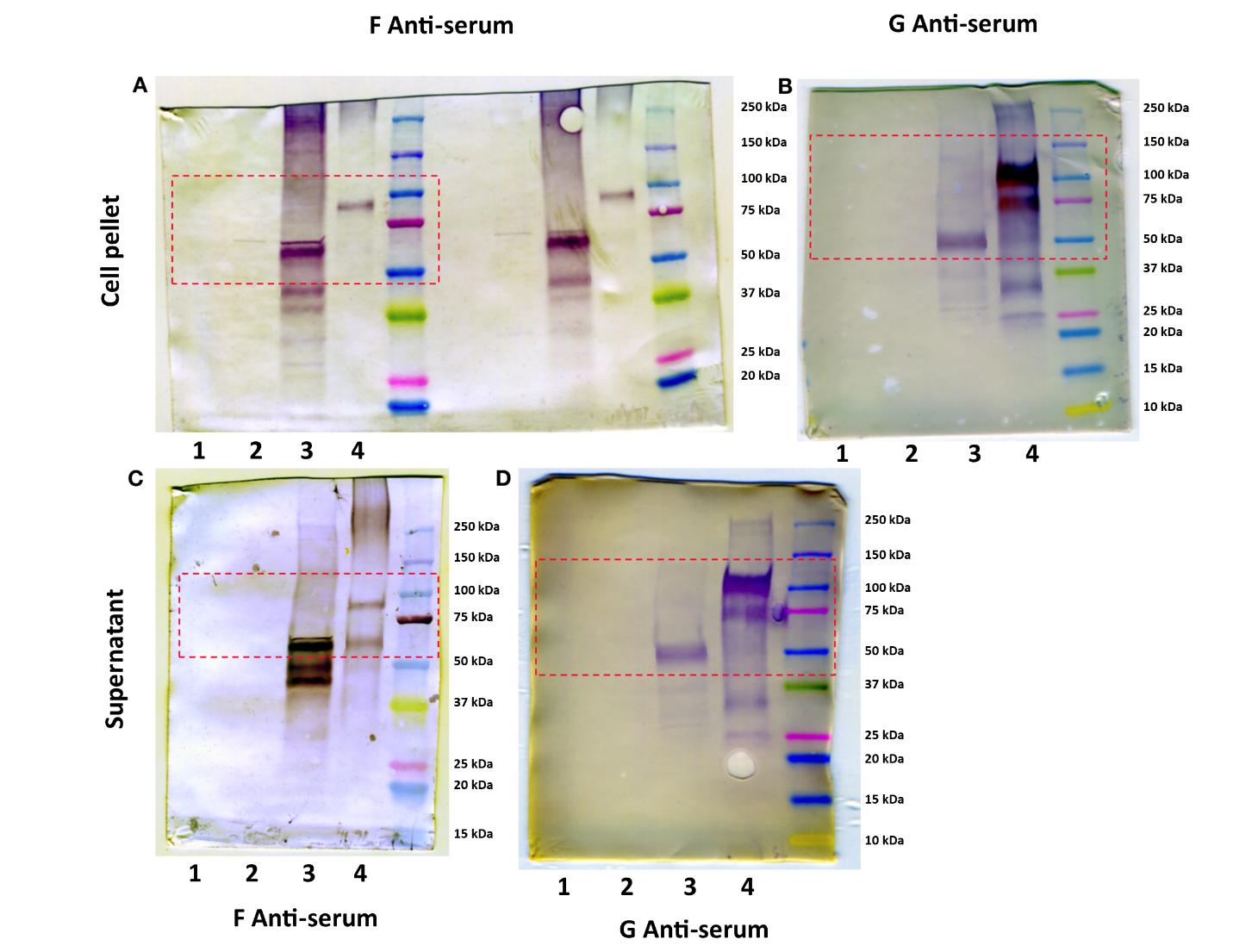


**Supplementary Figure 5.** Raw data for Supplementary Figure 3. Raw western blot images of cell pellet and supernatant samples taken from cells infected with RCN-FG. Western blots were cut prior to antibody hybridization to minimize blank space. Images A-D were cropped along the red-dotted boxes and converted to greyscale as seen in Supplementary Figure 3. **A)** Protein bands detected in Vero cell pellets using a fusion anti-serum. Red boxes outline the section cropped and displayed in Supplementary Figure 3A. Lane 1: Uninfected Vero cells. Lane 2: Vero cells infected with RCN-WT. Lane 3: Vero cells infected with RCN-FG. Lane 4: Purified NiV fusion protein (gF) exodomain (Native Antigen, Kidlington, Oxford, UK, SKU: REC31632). Lane 5: Uninfected Vero cells. Lane 6: Vero cells infected with RCN-WT. Lane 7: Vero cells infected with RCN-FG. Lane 8: Purified NiV fusion protein (gF) exodomain (Native Antigen, Kidlington, Oxford, UK, SKU: REC31632). **B)** Protein bands detected in Vero cell pellets using a glycoprotein anti-serum. Red boxes outline the section cropped and displayed in Supplementary Figure 3B. Lane 1: Uninfected Vero cells. Lane 2: Vero cells infected with RCN-WT. Lane 3: Vero cells infected with RCN-FG. Lane 4: Purified NiV fusion protein (gF) exodomain (Native Antigen, Kidlington, Oxford, UK, SKU: REC31632). **C)** Protein bands detected in the supernatant of Vero cells using a fusion anti-serum. Red boxes outline the section cropped and displayed in Supplementary Figure 3C. Lane 1: Uninfected Vero cells. Lane 2: Vero cells infected with RCN-WT. Lane 3: Vero cells infected with RCN-FG. Lane 4: Purified NiV fusion protein (gF) exodomain (Native Antigen, Kidlington, Oxford, UK, SKU: REC31632). **D)** Protein bands detected in the supernatant of Vero cells using a glycoprotein anti-serum. Red boxes outline the section cropped and displayed in Supplementary Figure 3D. Lane 1: Uninfected Vero cells. Lane 2: Vero cells infected with RCN-WT. Lane 3: Vero cells infected with RCN-FG. Lane 4: Purified NiV fusion protein (gF) exodomain (Native Antigen, Kidlington, Oxford, UK, SKU: REC31632).


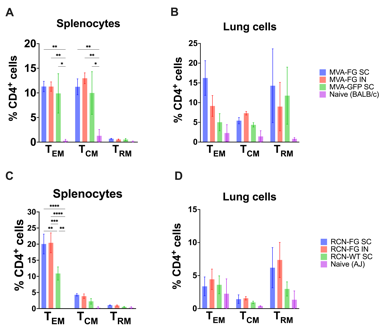


**Supplementary Figure 6.** CD4^+^ effector, central, and tissue-resident memory T cells. Phenotypic expression of CD4^+^ effector (CD62L^+^ CD44^-^), central (CD62L^+^ CD44^+^), and tissue-resident (CD103^+^ CD69^+^) memory T cells was evaluated in BALB/c and AJ mice 5 weeks post-boost. **A)** Splenocytes stimulated with F and G peptides from naive BALB/c and MVA-vaccinated mice. **B)** Lung cells stimulated with F and G peptides from naive BALB/c and MVA-vaccinated mice. **C)** Splenocytes stimulated with F and G peptides from naive AJ and RCN-vaccinated mice. **D)** Lung cells stimulated with F and G peptides from naive AJ and RCN-vaccinated mice. Data were represented with mean ± SEM with n = 1-4 for vaccination groups and n = 1 for naive mice. Samples were excluded based on poor cell recovery during tissue harvesting. Statistical analysis was conducted using a one-way ANOVA and a Tukey’s multiple comparison test after the discovery of significant differences.
